# Resveratrol Mitigates Age-Associated Cognitive Decline via Inhibition of cGAS-STING-Mediated Microglial Senescence

**DOI:** 10.3390/cells15060523

**Published:** 2026-03-16

**Authors:** Xinxin Duan, Jiahui Cheng, Jiayao Wang, Wen Chen, Zhi Ruan

**Affiliations:** School of Food Science and Engineering, South China University of Technology, Guangzhou 510641, China

**Keywords:** resveratrol, microglia, cGAS-STING signaling, cellular senescence, neuroinflammation, brain aging

## Abstract

**Background**: Aging-related cognitive decline is closely associated with microglial senescence and the resulting chronic neuroinflammation. Emerging evidence identifies the cyclic GMP-AMP synthase–stimulator of interferon genes (cGAS-STING) pathway as a pivotal innate immune signaling pathway linking DNA damage to cellular senescence and the senescence-associated secretory phenotype (SASP), particularly in microglia. Targeting the formation or selective clearance of senescent cells thus emerges as a promising therapeutic approach to ameliorate cognitive dysfunction. Resveratrol has shown promise in modulating immune response and exerting anti-aging effects. However, the therapeutic potential and underlying mechanisms of resveratrol in mitigating age-associated microglial senescence and cognitive decline are not fully understood. **Methods**: In the present study, we employed a well-established murine model of accelerated aging induced by chronic intraperitoneal injection of D-galactose (D-gal) to elicit pronounced senescence-associated phenotypes and neuroinflammation. Resveratrol was administered via oral gavage daily for three weeks following D-gal injections. Behavioral assays were conducted to assess cognitive performance. Immunohistochemistry, quantitative PCR, and Western blot analyses were used to evaluate markers of cellular senescence, microglial activation and pro-inflammatory cytokine expression. In addition, in vitro assays in cultured microglia coupled with RNA sequencing were used to investigate the downstream signaling events following resveratrol treatment. **Results**: Chronic D-gal treatment induced significant cognitive impairment, enhanced microglial activation, elevated pro-inflammatory cytokine levels, and increased markers of cellular senescence in the brain. Resveratrol administration remarkably attenuated these effects, as evidenced by improved memory performance, reduced microglial senescence markers, and suppressed expression of *Cxcl-10*, *Il-1β*, and other SASP factors. Mechanistically, unbiased transcriptomic analysis revealed that the cGAS-STING signaling and neuroinflammation pathways were prominently dysregulated with double-stranded DNA-induced cellular senescence, which was effectively normalized by resveratrol in cultured microglia. Interestingly, resveratrol inhibited the translocation of STING from the endoplasmic reticulum to the Golgi apparatus and suppressed phosphorylation of TBK1, thereby blocking downstream STING signaling. **Conclusions**: These findings demonstrate that resveratrol mitigates microglial senescence and neuroinflammation and preserves cognitive function in D-gal-induced aging mice, at least partly through modulation of the cGAS-STING signaling. Therefore, targeting this pathway may represent a promising therapeutic strategy for age-related neuroinflammatory and cognitive disorders.

## 1. Background

Aging is an inevitable biological process characterized by a progressive decline in tissue and organ function, and the brain is particularly vulnerable to age-related functional deterioration. With advancing age, cognitive abilities such as learning, memory, and executive function exhibit measurable decline [1,2], and aged individuals bear a substantially increased risk of developing neurodegenerative disorders, including Alzheimer’s disease (AD) and Parkinson’s disease (PD) [3]. These age-associated brain deficits are driven by a complex interplay of factors, including accumulative oxidative stress, chronic low-grade inflammation, DNA damage, mitochondrial dysfunction, and aberrant immune signaling [4,5]. Indeed, systemically chronic low-grade inflammation, often referred to as inflammaging, has been increasingly recognized as a fundamental hallmark of aging and a key contributor to age-related brain dysfunction [6,7]. The maintenance of brain homeostasis is dependent on tightly regulated immune and metabolic responses. Thus, failure of these processes leads to sustained neuroinflammation, synaptic dysfunction, and cognitive impairment [8].

Cellular senescence is a fundamental biological process defined as an essentially irreversible arrest of the cell cycle and is widely recognized as a hallmark of aging [9,10]. Senescent cells typically display elevated expression of cyclin-dependent kinase inhibitors, most notably *p16^Ink4a^* and *p21*^*Clip1*/*Waf1*^, which enforce growth arrest and distinguish senescent cells from quiescent states [11]. A defining molecular feature of senescent cells is the sustained activation of the DNA damage response, which promotes the formation of cytoplasmic chromatin fragments composed of genomic DNA and heterochromatin-associated proteins. After escaping the nucleus and accumulating in the cytosol, these self-DNA fragments potently activate innate immune signaling, thereby linking intracellular damage to inflammation and driving senescent phenotypes [12]. Central to this process is the cyclic GMP-AMP synthase–stimulator of interferon genes (cGAS-STING) pathway, an evolutionarily conserved DNA-sensing mechanism originally characterized for its role in antiviral immunity [13]. In aging tissues, persistent cytosolic DNA derived from damaged nuclei or mitochondria chronically activates cGAS, leading to STING-dependent transcriptional programs that include type I interferons and pro-inflammatory cytokines [14]. Increasing evidence demonstrates that dysregulated cGAS-STING signaling is a key driver of age-associated sterile inflammation and functional deterioration [13,15].

Microglia, the primary innate immune cells in the central nervous system, appear to be particularly vulnerable to aberrant activation of the cGAS-STING pathway [16]. Cytosolic DNA originating from damaged mitochondria or unrepaired nuclear DNA accumulates in aging microglia and directly engages cGAS, leading to the production of the second messenger 2′, 3′-cyclic GMP-AMP (2′, 3′-cGAMP). This molecule subsequently activates STING, triggering downstream transcriptional programs dominated by type I interferons and pro-inflammatory cytokines [17]. While transient activation of this pathway contributes to host defense, its chronic engagement in aging microglia promotes a sustained inflammatory state that is closely linked to senescence [18,19]. Studies in cultured microglia have demonstrated that DNA damage or mitochondrial dysfunction is sufficient to activate cGAS-STING signaling, resulting in reduced proliferative capacity, increased expression of senescence markers, and robust secretion of inflammatory mediators [20,21]. Importantly, genetic or pharmacological inhibition of cGAS or STING in these models attenuated the senescence-associated secretory phenotype (SASP) factor production and partially restored microglial homeostatic functions, indicating a causal role for this pathway in driving microglial senescence rather than a secondary consequence of inflammation [22]. In mouse models, activation of this pathway has been shown to mediate DNA damage-induced SASP and tissue inflammation in vivo, including within the brain [6,23]. Additionally, elevated markers of cGAS-STING activation have been detected in senescent human cells and aged tissues, including the brain, and correlate with inflammatory signatures associated with neurodegeneration [20,24]. Collectively, these data highlight cGAS-STING signaling as a central driver of microglial senescence and age-related neuroinflammation and support its therapeutic targeting as a strategy to mitigate neuroinflammation and cognitive decline associated with aging and neurodegenerative diseases.

Resveratrol is a naturally occurring non-flavonoid polyphenol present in more than 70 plant species [25]. In the central nervous system, resveratrol effectively suppresses neuroinflammatory responses, including attenuation of lipopolysaccharide-induced microglial activation and reduction in pro-inflammatory cytokine production in cultured microglia [26,27]. Consistent with these in vitro findings, in vivo studies in rodent models of neurological injury and neurodegenerative diseases showed that resveratrol limited microglial overactivation, ameliorated neuronal damage, and improved functional outcomes. Beyond its anti-inflammatory effects, accumulating evidence links resveratrol to the regulation of aging- and senescence-related processes. Resveratrol has been shown to alleviate peripheral and central inflammation in models of ischemic brain injury, reduce oxidative stress in aged animals with impaired antioxidant capacity, and preserve learning and memory performance in aging mice [28,29]. In AD transgenic models, resveratrol decreased microglial activation and dampened chronic neuroinflammation, a key contributor to disease progression [30]. Importantly, studies in cellular systems, including primary glial cultures and stem cell-derived neural cells, indicate that resveratrol modulates stress responses, DNA damage signaling, and inflammatory gene expression programs that are closely associated with cellular senescence [31]. Collectively, these findings suggest that resveratrol may influence not only acute inflammatory responses but also the long-term inflammatory states characteristic of microglial aging and senescence.

Despite these promising observations, the molecular mechanisms by which resveratrol influences microglial aging and senescence remain incompletely understood. In light of emerging evidence implicating the cGAS-STING pathway as a critical mediator of DNA damage-driven microglial senescence and inflammatory signaling in the aging brain, we hypothesized that resveratrol may exert its anti-inflammatory and neuroprotective effects, at least in part, through modulation of this pathway. To test this hypothesis, we combined transcriptomic profiling using RNA sequencing with in vivo animal models of aging to systematically investigate the impact of resveratrol on microglial senescence and cGAS-STING-dependent signaling. This integrated approach aimed to elucidate whether targeting the cGAS-STING signaling represents a mechanistic basis for the beneficial effects of resveratrol on microglial function and brain aging, thereby providing a rationale for its potential therapeutic application in age-related neuroinflammatory and neurodegenerative disorders.

## 2. Materials and Methods

### 2.1. Animals

Thirty-six male C57BL/6J mice, weighing 25 ± 2 g at seven weeks of age, were acquired from Jiangsu GemPharmatech Co., Ltd., Nanjing, China. Mice were housed in the specific pathogen-free (SPF) facility of the South China University of Technology Experimental Animal Center under standard conditions: room temperature 22–25 °C, relative humidity 50–60%, and a 12 h light/dark cycle. Standard chow and water were provided ad libitum. Following a 1-week acclimatization period, experimental interventions were initiated. All experimental procedures were reviewed and approved by the Animal Ethics Committee of the Experimental Animal Center, South China University of Technology (Protocol No. SPrz2024032). Mice were randomly allocated into three groups (n = 12 mice per group): control, model, and resveratrol-treated groups. Control animals received standard chow and were intraperitoneally injected with an equal volume of 0.9% sterile saline. The model and resveratrol groups were subjected to aging induction by daily intraperitoneal injection of D-galactose at 100 mg/kg for 8 consecutive weeks. Throughout the final 3 weeks, resveratrol-treated mice were given 50 mg/kg resveratrol daily by oral gavage. Mice in both the control and model groups were given an equivalent volume of 0.5% sodium carboxymethylcellulose (CMC-Na) solution by the same route and schedule.

### 2.2. Preparation of Resveratrol and D-Galactose Solution

Resveratrol was accurately weighed (50 mg) and dissolved in 10 mL of a 0.5% CMC-Na solution, which was prepared by dissolving 0.5 g of CMC-Na in 100 mL of 0.9% sterile saline. The mixture was thoroughly vortexed to produce a homogeneous suspension with a concentration of 5 mg/mL. The resveratrol suspension was freshly prepared each day and administered by oral gavage at a daily dose of 50 mg/kg body weight. To create a 10 mg/mL solution, D-galactose was dissolved in 0.9% sterile saline. Following membrane filtration for sterilization, the D-galactose solution was administered via intraperitoneal injection at a daily dose of 100 mg/kg body weight.

### 2.3. Behavioral Tests

All mice were pre-handled for 3 days prior to testing and habituated in the behavior room for 1 h before the test. All behavior tests were analyzed by the VisuTrack software 2.0 (Shanghai Xinruan Information Technology, Shanghai, China).

### 2.4. Open Field Test

The open field test was performed as follows. Briefly, the device was a 40 cm × 40 cm × 40 cm white acrylic arena that was custom-made [32]. Mice were placed at the periphery of the arena and allowed to freely explore for 5 min. Behavior was captured by a camera positioned above the arena. Total movement distance and time spent in the central area (10 cm × 10 cm) were automatically analyzed using software. After testing each mouse, the arena was cleaned with 75% ethanol.

### 2.5. Forced Alternation

The apparatus was a Y-shaped maze made of white acrylic [33]. Each arm was 30 cm long, 6 cm wide, and 15 cm high. Throughout the whole exam, visual cues were maintained in the testing room. In the initial test experiment, mice were allowed to visit two Y-maze arms for 5 min, while one arm was closed with a guillotine door. Mice were placed back in the start arm and given unrestricted access to all three arms for an additional 5 min following a 45 min inter-trial break. Each mouse was given a start and closed arm at random. Each mouse was tested, and then 75% ethanol was used to clear the maze. During the first 5 min of the second trial, we compared the average number of entries into the two familiar arms with the number of entries into the novel arm. VisuTrack software was used for scoring, and entries into an arm were counted when the nose point, center point, and tail base point for that arm were all within the defined area.

### 2.6. Rotarod Test

The rotarod test was performed as follows. Briefly, during the last three days of drug administration, mice were trained daily at fixed time points [34]. Mice were placed on the rotating rod for a 3 min habituation period at 4 rpm, followed by acceleration to the maximum speed (40 rpm) within 2 min. On the fourth day, the test was conducted with uniform acceleration from 4 rpm to 40 rpm over 2 min. Mice were allowed to remain on the device for a maximum of 5 min. Latency to fall and total walking distance were recorded. Each mouse was tested at least three times daily, with at least 1 h between trials. After testing each mouse, the rotarod apparatus was cleaned with 75% ethanol.

### 2.7. Histological Processing and Immunochemistry Staining

Brain tissues were removed after transcardial perfusion fixation with phosphate-buffered saline (PBS), followed by post-fixation with 4% paraformaldehyde (Beyotime, #P0099, Shanghai, China) and cryoprotection with 30% sucrose/PBS. Fixed frozen brain tissues were coronally sectioned using a cryostat (Leica CM1950, Nussloch, Germany) at 30 µm thickness, and three sections of dorsal hippocampal regions per brain per antibody were used for immunochemistry. The sections were subjected to antigen retrieval with sodium citrate (10 mM, pH 6.0) at 95 °C for 20 min, then blocked in 5% normal donkey serum, 5% bovine serum albumin (BSA, Millipore Sigma, #A1933, Burlington, MA, USA) and 0.1% TritonX-100/PBS for 1 h, followed by incubation with primary antibodies (pTBK1, 1:100, #5483S, Cell Signaling Technology, Danvers, MA, USA; IBA1, 1:1000, #019-19741, Wako, Tokyo, Japan; P2ry12, 1:100, #848002, Biolegend, CA, USA; TUB33, 1:500, #66375-1-lg, Proteintech, Wuhan, China; CD68, 1:100, #MCA1957, Bio-Rad, Hercules, CA, USA; β-gal, 1:200, #15518-1-AP, Proteintech, Wuhan, China), diluted with 1% BSA and 0.02% tween in PBS overnight. The samples were washed with PBST 3 times and incubated in secondary antibodies (donkey anti-rabbit IgG AlexaFluor 594, 1:2000, Thermo Fisher Scientific, #A32754, Waltham, MA, USA; donkey anti-mouse IgG AlexaFluor 488, 1:2000, Thermo Fisher Scientific, #A32766, Waltham, MA, USA) for 2 h at room temperature in darkness. After PBST washing, DAPI (Beyotime, #C1006) staining solution was added and incubated for 3 min at room temperature in the dark. Sections were mounted with anti-fade mounting medium. Subsequently, images were observed and captured using a fluorescence or confocal microscope and analyzed with the ImageJ image analysis system.

### 2.8. Confocal Image Processing and Quantification

All confocal imaging was performed on a Nikon A1 using NIS-Elements C software 4.0 (Nikon, Tokyo, Japan) at the inverted microscope stand using the confocal mode with 63×/1.4 oil immersion objectives. Images of 1024 × 1024 pixels were acquired as confocal stacks with a z-interval of 20 μm using a system-optimized setup to image cells. For imaging IBA1, β-gal, pTBK1 and STING, a 552 nm laser line was used, and emission was collected at 565–650 nm; for imaging CD68, P2ry12, GM130, and TUB33, a 488 nm laser line was used, and emission was collected at 490–600 nm. All co-localization images were scanned frame by frame in the sequential scanning mode, which showed no cross-talk among multiple channels. Gain and off-set were set at values that prevented saturated and empty pixels. After image acquisition, all images were applied with lightning deconvolution. ImageJ was used to perform background subtraction, thresholding, and quantification. Final data analysis was performed using Microsoft Excel, and graph rendering was performed in GraphPad Prism 10.0.

### 2.9. SA-β-Gal Staining

SA-β-galactosidase staining was performed on fresh-frozen tissue sections according to established protocols with minor modifications (Cell Signaling Technology, #9860). Briefly, 30 μm cryosections were thawed and washed with PBS. After three washes with PBS, sections were incubated in SA-β-gal staining solution consisting of 1 mg/mL X-Gal, 5 mM potassium ferricyanide, 5 mM potassium ferrocyanide, and 2 mM MgCl_2_ in citric acid-sodium phosphate buffer (pH 6.0). Incubation was carried out at 37 °C for 24 h in a humidified chamber protected from light. Tissue sections were then rinsed with PBS and examined under a bright-field microscope. HMC-3 cells were stained in the same SA-β-gal staining solution at 37 °C for 24 h following the instructions. Senescence-associated β-galactosidase activity was visualized as an insoluble blue precipitate within cells. For double-labeling experiments, immunohistochemical staining was subsequently performed on the same sections following SA-β-gal staining. All staining steps were performed simultaneously for comparative experimental groups to minimize inter-assay variability.

### 2.10. Western Blot

Western blot analyses were performed as follows. Cells and tissues from each group were lysed using Western IP lysis buffer (Beyotime, #P0013, Shanghai, China) to extract total proteins. Protein concentrations were measured with the BCA Pierce Protein Assay Kit (Thermo Fisher Scientific, #23225, Waltham, MA, USA) and normalized to the lowest concentration. Proteins were separated by SDS-PAGE and transferred to nitrocellulose membranes. Membranes were blocked with 5% non-fat milk for 1 h at room temperature, followed by incubation with primary antibodies (TBK1, 1:2000, #3504S; pTBK1, 1:1000, #5483S; STING, 1:1000, #13647S; pSTING, 1:1000, #62912S; GAPDH, 1:10,000, #2118S, Cell Signaling Technology) in antibody dilution buffer overnight at 4 °C. Secondary antibodies (anti-mouse or anti-rabbit HRP-conjugated) were applied for 1 h at room temperature. Protein bands were visualized using enhanced chemiluminescence substrate and imaged with a ChemiDoc XRS BioRad (BioRad, Hercules, CA, USA) imaging system with Image Lab software 6.1. Antibodies used are listed in the Supplementary Table S1.

### 2.11. Cell Culture

Murine microglial cell line BV-2 and human microglial cell line HMC-3 were purchased from Shanghai Cell Bank, Chinese Academy of Sciences. BV-2 cells were cultured in DMEM basal medium, while HMC-3 cells were maintained in MEM basal medium. Both media were supplemented with 10% (*w*/*v*) fetal bovine serum (FBS) and 1% penicillin/streptomycin. Cells were incubated at 37 °C in a humidified atmosphere of 5% CO_2_/95% air (*v*/*v*).

### 2.12. Preparation and Transfection of dsDNA

The dsDNA probes were generated by annealing complementary single-stranded DNA oligonucleotides (sense: ACATCTAGTACATGTCTAGTCAGTATCTAGTGATTATCTAGACATACATGATCTATGACATATATAGTGGATAAGTGTGG; anti-sense: CCACACTTATCCACTATATATGTCATAGATCATGTATGTCTAGATAATCACTAGATACTGACTAGACATGTACTAGATGT) [35]. The annealing reaction was assembled in a total volume of 100 µL, comprising 20 µL of 5× annealing buffer, 100 µM of each oligonucleotide (sense and anti-sense), and nuclease-free PCR-grade water. The mixture was subjected to a stepwise thermal annealing protocol as follows: 95 °C for 4 min, 85 °C for 4 min, 82 °C for 4 min, 78 °C for 4 min, 75 °C for 4 min, 72 °C for 4 min, 70 °C for 10 min, followed by a gradual cooling step at 69 °C with a −1 °C per cycle ramp for 58 cycles (1 min per cycle), and a final hold at 10 °C for 1 min. Cells were transfected with dsDNA using the jetPRIME^®^ (Polyplus, #101000046, Strasbourg, France) transfection reagent according to the manufacturer’s instructions. Briefly, cells were seeded in 6-well plates at a density of 300,000 cells per mL in DMEM supplemented with 10% FBS and cultured for 24 h at 37 °C under 5% CO_2_. Prior to transfection, the medium was replaced with 1 mL of fresh complete medium per well. For each well, 2 µg of annealed dsDNA was diluted in jetPRIME^®^ buffer to a total volume of 100 µL. As a negative control, an equivalent volume of 1× annealing buffer was used in place of dsDNA. Subsequently, 2 µL of jetPRIME^®^ reagent was added to each mixture, vortexed briefly, and incubated at room temperature for 10 min. The transfection complex was then added dropwise to the cells. After gentle swirling to ensure even distribution, cells were incubated at 37 °C in a humidified atmosphere containing 5% CO_2_ for the duration required by each experiment.

### 2.13. Compound Treatment

Resveratrol and H-151 stock solutions were prepared in dimethyl sulfoxide (DMSO) at 0.7 mM or 1 mM, respectively, and stored at −20 °C. Working solutions of resveratrol (0.7 µM) and H-151 (1 µM) were freshly prepared in DMEM prior to use. Control cells received equivalent volumes of DMSO in all experiments. Cells were pre-treated with resveratrol or H-151 for 1 h. Following pre-treatment, dsDNA was added and cells were co-treated for 9 h with the original compounds present.

### 2.14. Immunofluorescence Staining

Cells were fixed with 4% paraformaldehyde for 15 min, permeabilized with 0.1% Triton X-100 for 10 min, and washed with PBS. After blocking with 5% bovine serum albumin for 1 h, cells were incubated with primary antibodies (pTBK1, 1:100, #5483S, Cell Signaling Technology; pSTING, 1:100, #62912S, Cell Signaling Technology; STING, 1:200, #19851-1-AP, Proteintech; GM130, 1:200, #610822, BD Bioscience, Franklin Lakes, NJ, USA; P21, 1:100, #67362-1-Ig, Proteintech) overnight at 4 °C. Following PBS washes, samples were incubated with fluorescent secondary antibodies for 1 h, counterstained with DAPI, and visualized using an inverted fluorescence microscope.

### 2.15. ELISA

Cell culture supernatants were collected and centrifuged at 500× *g*, 4 °C to remove cell debris and dead cells. TNF-α secreted in supernatants was measured using a mouse TNF-α ELISA kit (Invitrogen, #BMS607-3, Carlsbad, CA, USA) according to the manufacturer’s instructions. Cells were washed with ice-cold PBS and lysed in RIPA buffer (Beyotime, #P0013B) containing 1X protease and phosphatase inhibitor cocktail (Beyotime, #P1045). Lysates were clarified by centrifugation, and protein concentration was determined using the Pierce™ BCA Protein Assay Kit (Thermo Fisher Scientific, #23225, Waltham, MA, USA).

### 2.16. Real-Time Quantitative PCR

Total RNA was isolated from frozen hippocampus and cortex tissue or from cells with Trizol (Invitrogen, #15596018CN, Carlsbad, CA, USA) and was reverse transcribed using a Master Mix kit (TaKaRa, #RR092S, Kyoto, Japan), and the resulting complete DNA (cDNAs) were diluted 10-fold before use. To evaluate the level of gene expression, real-time quantitative PCR (RT-qPCR) with SYBR Green dye was applied. PCR reactions were set up in an 8-well PCR tube strip with a 10 μL final volume. The reaction mixture contained 5 μL SYBR™ Green PCR Master Mix (TaKaRa, #CN830S), 10 μM of each primer (forward and reverse) and 1 μL cDNA. All samples were run in duplicate. Amplification was carried out for 39 cycles with the standard program according to the manufacturer’s specifications in a CFX Opus96 RT-qPCR machine. Relative amounts of mRNA were calculated as the comparative C*_t_* after normalization to the GAPDH control C*_t_* value.

### 2.17. Construction of RNA Sequencing Libraries and Sequencing

The Trizol reagent (Invitrogen, #15596018CN) was used separately to extract total RNA from the cell samples. Agilent 2200 was used to assess the RNA quality, which was stored at −80 °C. RNA with an RNA integrity number (RIN) greater than 7.0 was used to generate cDNA libraries. The VAHTS Universal V6 RNA-seq Library Prep Kit for Illumina (vazyme, Inc., Nanjing, China) was used to create the cDNA libraries for every RNA sample in accordance with the guidelines provided by the manufacturer (Shanghai NovelBio Pharmaceutical Technology Co., Ltd., Shanghai, China). In general, the following phases make up the protocol: Using oligo(dT) magnetic beads, poly-A-containing mRNA was isolated from 1 μg of total RNA. It was then broken up into 200–600 bp using divalent cations at 85 °C for 6 min. First- and second-strand cDNA synthesis was carried out using the cleaved RNA fragments. Second-strand cDNA synthesis, which permits the removal of the second strand, was carried out using dUTP mix. The cDNA segments were ligated using indexed adapters, end repaired, and A-tailed. Uracil DNA glycosylase was used to purify the ligated cDNA products and eliminate the second-strand cDNA. The cDNA libraries were produced by PCR enrichment of purified first-strand cDNA. Agilent 2200 was used for quality control, and DNBSEQ-T7 was used for a 150 bp paired-end run of sequencing.

### 2.18. RNA Sequencing Mapping

Prior to read mapping, adaptor sequences and low-quality reads were eliminated from the raw reads to provide clean reads. The Star was then used to align the clean reads to the mouse genome (mm10, Ensembl100). Gene counts were obtained using HTseq, and gene expression was ascertained using the RPKM technique.

### 2.19. Analysis of Differentially Expressed Genes

We applied the DESeq2 algorithm to filter the differentially expressed genes, after the significant analysis, *p*-values and FDR analysis were subjected to the following criteria: (i) fold change > 2 or <0.5; (ii) FDR < 0.05.

### 2.20. GO Analysis

To further clarify the biological consequences of the experiment’s differentially expressed genes, gene ontology (GO) analysis was carried out. The GO annotations were obtained from Gene Ontology (http://www.geneontology.org/), UniProt (http://www.uniprot.org/), and NCBI (http://www.ncbi.nlm.nih.gov/). Fisher’s exact test was used to determine which GO categories were significant (*p*-value < 0.05).

### 2.21. Pathway Analysis

To determine the significant route of the genes that were differentially expressed based on the KEGG database, pathway analysis was employed. To choose the significant pathway, we used Fisher’s exact test, and a *p*-value of less than 0.05 was used to set the significance threshold.

### 2.22. Statistical Analysis

All data in graphs are presented as the mean ± standard error of the mean (SEM). Statistical analyses were performed using GraphPad Prism (GraphPad Software, Boston, MA, USA). Two-group comparisons were made using a two-tailed unpaired Student’s *t*-test. Multiple comparisons were performed by either one- or two-way ANOVA, followed by Dunnett’s or Sidak’s multiple comparisons test used as indicated in the figure legends and methods section. A statistically significant difference was assumed at *p* < 0.05. For differential expression analysis of transcriptomic data, significance was set as an FDR-corrected *p*-value (*q*-value).

## 3. Results

### 3.1. Resveratrol Improved the Cognitive Function of Aging Mice

**Figure 1:**
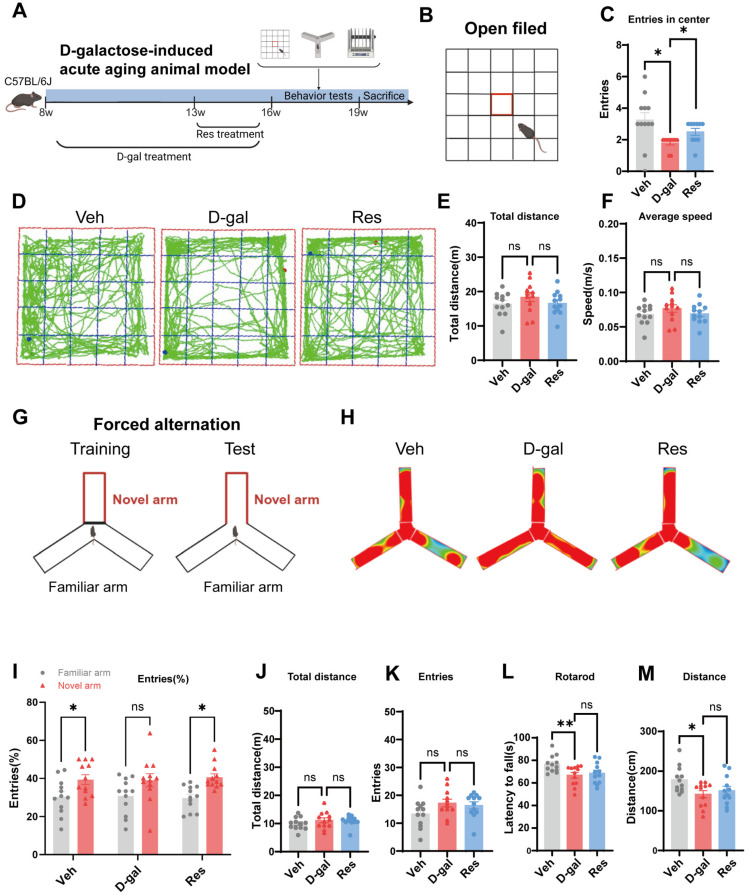
Resveratrol enhanced cognitive and behavioral performance in aging mice. (**A**) Schematic diagram of the establishment of the aging model, resveratrol administration schedule, and subsequent behavioral assessments. (**B**) Diagram of the open field test apparatus and procedure. (**C**) Quantification of total entries into the central zone in the open field test. (**D**) Representative locomotor trajectories of mice during the open field test. (**E**,**F**) Total distance traveled (**E**) and average speed (**F**) recorded in the open field test. (**G**) Schematic diagram of the forced alternation test. (**H**) Representative trajectory plots of mouse exploration in the forced alternation task. (**I**) Percentage of entries into the novel arm relative to the familiar arm. (**J**,**K**) Total distance traveled (**J**) and total number of arm entries (**K**) during the forced alternation test. (**L**,**M**) Latency to fall (**L**) and distance traveled (**M**) in the rotarod test. N = 12 mice per group. All the bar graphs are represented by the mean ± SEM; statistical significance was performed with one-way or two-way ANOVA followed by Dunnett’s or Sidak’s multiple comparisons test, * *p* < 0.05, ** *p* < 0.01; ns, not significant. Veh, vehicle; D-gal, D-galactose; Res, resveratrol.

To determine the effects of resveratrol on aging-associated cognitive decline, we established an acute aging animal model by intraperitoneally injecting 8-week-old male C57BL/6J mice with D-galactose (D-gal, 100 mg/kg/day) for 8 consecutive weeks [36]. The D-gal model is widely used to induce accelerated brain aging in rodents. D-gal accumulates intracellularly when administered at high doses, overwhelming normal metabolism and generating excessive reactive oxygen species via galactose oxidation and advanced glycation end products. Sustained metabolic and oxidative stress activates senescence pathways (e.g., p21, p16), and promotes SASP, collectively leading to cognitive decline and other hallmarks of brain aging [37]. Beginning in the 6th week of D-gal administration, resveratrol was delivered by oral gavage. Throughout the treatment period, no significant change in body weight was observed in the animals (Supplementary Figure S1). After 3 weeks of resveratrol treatment the, cognitive and motor functions of those animals were evaluated. Behavioral assessments included open field, forced alternation, and rotarod assays (Figure 1A). At 16 weeks of age, we conducted an open field test to assess spontaneous locomotion in aging mice with or without resveratrol treatment (Figure 1B). The open field test is a standard assay that examines spontaneous locomotor activity and anxiety-like behavior in rodents. It exploits the innate conflict between exploratory drive and avoidance of exposed areas; rodents normally prefer to remain near walls and avoid the central zone, which is interpreted as an index of anxiety-like behavior in a novel open environment [38,39]. In this experiment, we quantified anxiety-related exploratory behavior using the number of central square crossings. Interestingly, resveratrol-treated aged mice demonstrated a pattern of exploration more similar to that of vehicle-injected mice, with a greater tendency to enter and traverse the central squares compared with untreated aged mice (Figure 1C,D). No differences were observed in total distance traveled or average speed among the three groups within the arena, suggesting that drug treatment did not affect spontaneous locomotor activity in this test (Figure 1E,F). Subsequently, the forced alternation task was used to evaluate spatial recognition memory (Figure 1G). Interestingly, the animal travel heatmaps revealed differential exploration behaviors among groups (Figure 1H). D-gal-induced aging animals showed no preference to enter the new arm when compared to vehicle-injected mice, suggesting a recognition memory deficit (Figure 1I). On the other hand, mice given resveratrol showed a preference for the new arm over the ones they knew, indicating that functional recognition memory had been restored (Figure 1I). It suggested that resveratrol could enhance the spatial working memory of aging animals. There was no significant difference observed among groups in total arm entries or total distance traveled, indicating that D-gal or drug treatment did not affect the free exploration activity of those animals in this experiment (Figure 1J,K). In addition, to evaluate the motor function of those mice, rotarod tests were performed. The rotarod assay is a widely accepted method for assessing motor coordination, balance, and neuromuscular performance in rodents [40,41]. It measures the ability of an animal to maintain position on a rotating rod, which reflects integrated motor control and endurance. In our study, intraperitoneal injection of D-gal significantly reduced both the latency to fall and the distance traveled on the rotarod compared with controls. This decline indicates impaired motor coordination and balance in the aging model. Mice treated with resveratrol showed a trend toward increased time and distance on the rotarod compared to the D-gal-induced aging mice (Figure 1L,M). Therefore, the above behavioral results suggested that resveratrol could ameliorate the cognitive and behavioral dysfunction induced by aging.

### 3.2. Resveratrol Suppressed Microglial Senescence in Aging Mice

**Figure 2:**
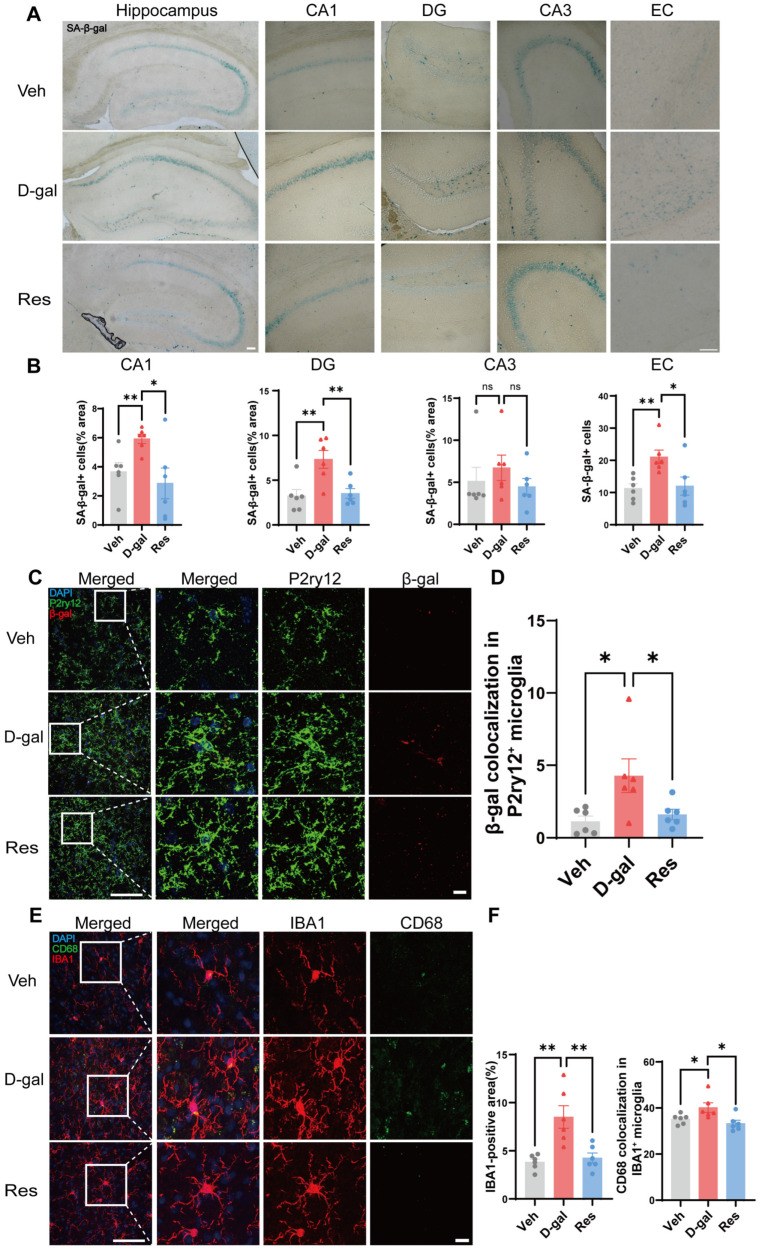
Inhibition of microglial senescence by resveratrol in aging mouse brains. (**A**) Representative SA-β-gal staining of brain sections from Veh, D-gal or resveratrol-treated mice, scale bar = 100 μm. (**B**) Quantification of SA-β-gal staining intensity of CA1, DG, CA3 subregions of hippocampus and entorhinal cortex. (**C**) Representative immunofluorescence images of microglial marker P2ry12 (green) and senescence marker β-gal (red) in brain sections from Veh, D-gal or Res-treated aging mice, n = 6 mice per group, scale bars = 50 μm (left) or 10 μm (right). (**D**) Quantification of β-gal staining intensity colocalized with P2ry12. (**E**) Representative immunofluorescence images of microglial marker IBA1 (red) and activated microglia marker CD68 (green) in brains of Veh, D-gal or Res-treated mice, scale bars = 50 μm (left) or 10 μm (right). (**F**) Quantification of IBA1-positive area and CD68 intensity colocalized with IBA1. N = 6 mice per group. All the bar graphs are represented by mean ± SEM; statistical significance was performed with one-way ANOVA followed by Dunnett’s multiple comparisons test, * *p* < 0.05, ** *p* < 0.01; ns, not significant. Veh, vehicle; D-gal, D-galactose; Res, resveratrol; CA1, cornu ammonis 1; DG, dentate gyrus; CA3, cornu ammonis 3; EC, entorhinal cortex.

To assess the effects of resveratrol on brain aging, senescence-associated β-galactosidase (SA-β-gal) staining was performed in mouse brain sections. Elevated SA-β-gal activity is a well-established marker of cellular senescence. In the present study, D-gal-injected mice showed a marked increase in SA-β-gal staining in the hippocampal cornu ammonis 1 (CA1) and dentate gyrus (DG) subregions, as well as in the cerebral cortex, compared with vehicle-injected controls. Interestingly, resveratrol administration significantly reduced the SA-β-gal staining in both the hippocampus and cortex of aging mice (Figure 2A,B). This result suggests that resveratrol alleviated brain senescence induced by D-gal. Because oxidative DNA damage contributes to cellular senescence, we next examined 8-hydroxy-2′-deoxyguanosine (8-OHdG), a widely used marker of oxidative DNA damage. Elevated 8-OHdG levels were observed in the D-gal group, whereas resveratrol markedly reduced oxidative DNA damage (Supplementary Figure S2A,B), suggesting that the anti-senescence effect of resveratrol may involve suppression of oxidative stress. Accumulation of oxidative DNA lesions such as 8-OHdG is known to increase during brain aging. To evaluate cell-type-specific senescence, we assessed microglia as key immune regulators in brain aging. Confocal co-localization staining of the microglial marker P2ry12 with SA-β-gal showed enhanced β-galactosidase activity in microglia of D-gal-treated mice (Figure 2C). Resveratrol treatment significantly lowered this signal (Figure 2C,D). Consistently, co-staining of IBA1 with the senescence marker p21 further confirmed enhanced microglial senescence in the D-gal group and its attenuation following resveratrol treatment (Supplementary Figure S2C,D). Notably, supplementary data showed that neuronal senescence markers did not change significantly in resveratrol-treated mice (Supplementary Figure S2E). Collectively, these findings highlight that D-gal predominantly affects microglial aging in this experimental paradigm, and resveratrol preferentially attenuates microglial senescence rather than neuronal senescence under these conditions. In addition, we further assessed microglial activation using IBA1 and CD68 co-staining. The number of IBA1-positive microglia and CD68 signal intensity were significantly increased in D-gal-treated mice, consistent with microglial overactivation (Figure 2E). Resveratrol reduced both microglial numbers and CD68 expression (Figure 2E,F), supporting its role in suppressing microglial activation. Collectively, these results indicated that intraperitoneal injection of D-gal could induce a senescent state in microglia in the mouse brain, and resveratrol treatment could suppress the microglial activation and senescence.

### 3.3. Resveratrol Reduced Senescence and Inflammatory Responses in Microglial Cells

**Figure 3:**
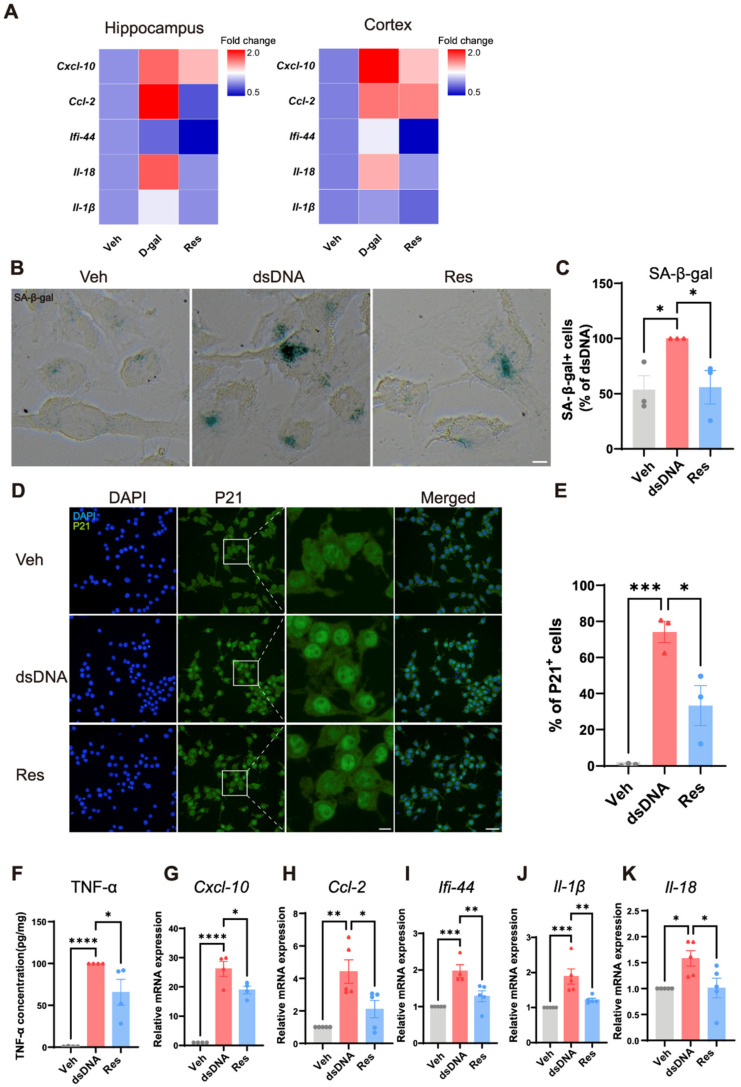
Resveratrol reduced senescence-associated secretory phenotype and inflammation response. (**A**) RT-qPCR analysis of mRNA expression levels of *Cxcl-10*, *Ccl-2*, *Ifi-44*, *Il-1β*, and *Il-18* in the cerebral cortex and hippocampus of mice, n = 6 mice per group. (**B**) Representative SA-β-gal staining of human microglia HMC-3 cells treated with resveratrol, scale bar = 10 μm. (**C**) Quantification of SA-β-gal staining intensity in HMC-3 cells, n = 3 biological replicates, data are represented as mean ± SEM; statistical significance was performed with one-way ANOVA followed by Dunnett’s multiple comparisons test, * *p* < 0.05. (**D**) Representative immunofluorescence images of P21 in BV-2 cells, scale bars = 10 μm (left) or 50 μm (right); (**E**) Quantification of P21 positive cells in BV-2 cells, n = 3 biological replicates, data are represented as mean ± SEM; statistical significance was performed with one-way ANOVA followed by Dunnett’s multiple comparisons test, * *p* < 0.05, *** *p* < 0.001. (**F**) TNF-α protein level in culture supernatants of murine microglia BV-2 cells, measured by ELISA, n = 4 biological replicates, data are represented as mean ± SEM; statistical significance was performed with one-way ANOVA followed by Dunnett’s multiple comparisons test, * *p* < 0.05. (**G**–**K**) RT-qPCR analysis of mRNA expression levels of *Cxcl-10* (**G**), *Ccl-2* (**H**), *Ifi-44* (**I**), *Il-1β* (**J**), and *Il-18* (**K**), in BV-2 cells, n = 4–5 biological replicates, data are represented as mean ± SEM; statistical significance was performed with one-way ANOVA followed by Dunnett’s multiple comparisons test, * *p* < 0.05, ** *p* < 0.01, *** *p* < 0.001, **** *p* < 0.0001. Veh, vehicle; D-gal, D-galactose; Res, resveratrol; dsDNA, double-stranded DNA; SA-β-gal, senescence-associated beta-galactosidase.

To further investigate the effects of resveratrol on neuroinflammation in aging mice models, we selected to measure several key SASP factors, including *Cxcl-10*, *Ccl-2*, *Ifi-44*, *Il-1β*, and *Il-18*. We evaluated SASP expression in both the cortex and the hippocampus of aging mice and resveratrol-treated mice. RT-qPCR analysis showed that D-gal-induced aging mice exhibited elevated transcripts of *Cxcl-10*, *Ccl-2*, *Ifi-44*, *Il-1β*, and *Il-18* (Figure 3A). Interestingly, resveratrol effectively suppressed the expression of these SASP factors in both brain regions, indicating suppression of age-related inflammatory signaling (Figure 3A). Next, to determine whether resveratrol effects could be recapitulated at the cellular level, microglial cells were transfected with double-stranded DNA (dsDNA) to induce senescence. Cytosolic dsDNA acts as a trigger of cellular senescence because it activates the innate cytoplasmic DNA-sensing pathway, leading to inflammatory signaling, and promotes key features of cellular senescence, including SASP factor expression [42]. After dsDNA exposure, optical microscopy and quantitative analysis revealed a marked increase in SA-β-gal activity, confirming induction of senescence (Figure 3B,C). Importantly, compared to dsDNA alone, resveratrol therapy significantly decreased the percentage of SA-β-gal-positive cells (Figure 3B,C). Consistent with these findings, immunofluorescence analysis of the senescence marker p21 showed that dsDNA treatment markedly increased the nuclear accumulation of p21. In contrast, resveratrol treatment significantly attenuated this dsDNA-induced nuclear accumulation of p21 (Figure 3D,E). We then examined the expression of SASP factors in the microglial cell model. dsDNA treatment significantly upregulated pro-inflammatory mediators, including the protein level of TNF-α (Figure 3F), and mRNA expression of *Cxcl-10*, *Ccl-2*, *Ifi-44*, *Il-1β*, and *Il-18* (Figure 3G–K). Resveratrol effectively inhibited this SASP induction, restoring lower expression levels of these cytokines and chemokines (Figure 3G–K). These findings thus further support that resveratrol reduced both senescence and associated inflammatory responses in microglial cells.

### 3.4. Transcriptomic Profiling Revealed cGAS-STING Pathway Alterations Following Resveratrol Treatment

**Figure 4:**
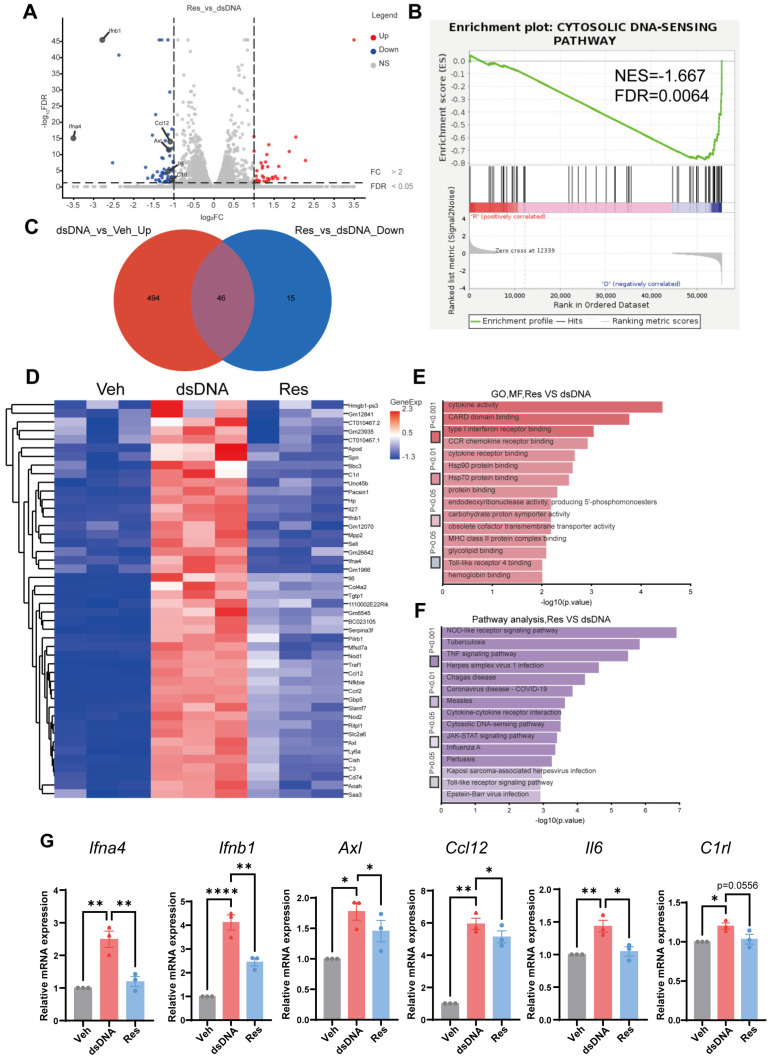
RNA sequencing analysis uncovered cGAS-STING pathway alterations following resveratrol treatment. (**A**) Selection of DEGs that were downregulated by Res or upregulated by dsDNA and associated with key modules related to cellular senescence progression or the cGAS-STING pathway. (**B**) GSEA showed downregulation of the Cytosolic DNA-Sensing Pathway in Res-treated BV-2 cells compared with dsDNA-treated controls. (**C**) Venn diagram illustrated overlapping DEGs between Res-downregulated and dsDNA-upregulated gene sets. (**D**) Heatmap of RNA sequencing data depicting expression profiles of indicated genes in BV-2 cells. (**E**,**F**) Functional enrichment analyses, GO analysis showed alterations in molecular function in Res-treated BV-2 cells (**E**); KEGG analysis indicated suppression of major pathways in Res-treated BV-2 cells (**F**). (**G**) Validation of selected cGAS-STING pathway-related and inflammation-related DEGs by RT-qPCR in BV-2 cells, n = 3 biological replicates. All the bar graphs are represented by mean ± SEM; statistical significance was performed with one-way ANOVA followed by Dunnett’s multiple comparisons test, * *p* < 0.05, ** *p* < 0.01, **** *p* < 0.0001. Veh, vehicle; dsDNA, double-stranded DNA; Res, resveratrol; DEGs, differentially expressed genes; GSEA, gene set enrichment analysis; KEGG, Kyoto Encyclopedia of Genes and Genomes; GO, gene ontology.

To systematically explore the cellular phenotypes associated with resveratrol inhibition of STING, we performed RNA sequencing analysis in BV-2 cells. Differentially expressed genes (DEGs) analysis between the dsDNA group and the resveratrol-treated group revealed widespread transcriptomic changes. In total, 540 genes were highly expressed in the dsDNA group, whereas 61 genes were significantly downregulated following resveratrol treatment. Genes associated with the Cytosolic DNA-Sensing Pathway and neuroinflammation were annotated (Figure 4A). Gene set enrichment analysis (GSEA) revealed significant activation of immune and DNA-sensing pathways in the dsDNA group. Enriched signatures included Allograft Rejection (NES = 1.678, FDR < 0.001), Graft-versus-Host Disease (NES = 1.680, FDR < 0.001), and Cytosolic DNA-Sensing Pathway (NES = 1.660, FDR < 0.001). Additional activation of the RIG-I-Like Receptor Signaling Pathway and Autoimmune Thyroid Disease was also observed (Supplementary Figure S3A,B). Conversely, resveratrol treatment markedly suppressed these pathways. Notably, Allograft Rejection (NES = −1.689, FDR = 0.0048), Graft-versus-Host Disease (NES = −1.713, FDR = 0.0026), and the Cytosolic DNA-Sensing Pathway (NES = −1.667, FDR = 0.0064) were downregulated following resveratrol treatment (Figure 4B; Supplementary Figure S3C). Pathways involved in antigen processing and presentation also showed reduced enrichment. Given that cGAS and STING function as core sensors of cytosolic DNA and drive downstream immune signaling, the suppression of DNA-sensing signatures by resveratrol suggests targeted modulation of this pathway. Recent studies have established that cGAS-STING activation occurs in response to cytosolic DNA accumulation, DNA damage, and neuroinflammation [6]. Furthermore, a Venn diagram identified 46 overlapping DEGs between comparisons (Figure 4C). These overlapping genes were visualized in a clustered heatmap to illustrate expression patterns (Figure 4D). To further characterize the functional relevance of these genes, we performed gene ontology (GO) and Kyoto Encyclopedia of Genes and Genomes (KEGG) enrichment analyses on the 46 overlapping genes. GO analysis showed that molecular functions were enriched for cytokine activity, CARD domain binding, and type I interferon receptor binding (Figure 4E). Biological processes included response to bacterium, immune system processes, and inflammatory response. Enriched cellular components were the extracellular space, the extracellular region, and the external side of the plasma membrane (Supplementary Figure S3D–G). KEGG pathway analysis highlighted significant enrichment in the TNF signaling pathway and the Cytosolic DNA-Sensing Pathway, further linking the overlapping gene set to immune signaling and DNA recognition (Figure 4F). To validate transcriptomic findings, selected genes related to the Cytosolic DNA-Sensing Pathway and neuroinflammation were assessed by RT-qPCR in BV-2 cells. These validations confirmed the expression trends observed in RNA-seq (Figure 4G). Collectively, these data indicate that resveratrol markedly alters gene expression programs associated with the cGAS-STING signaling pathway. The results support a model in which resveratrol attenuates cellular senescence and neuroinflammatory signaling by suppressing cytosolic DNA-sensing and related immune pathways.

### 3.5. Resveratrol Prevented cGAS-STING Pathway Activation in the Mouse Brain

**Figure 5:**
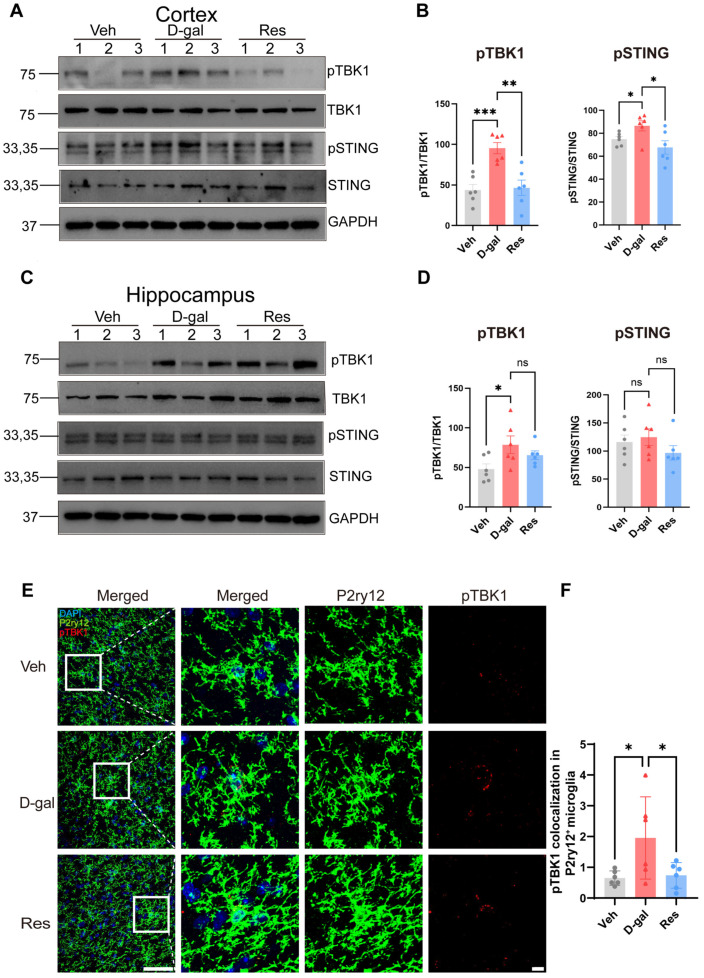
Resveratrol prevented excessive activation of the cGAS-STING pathway in aging mouse brains. (**A**,**C**) Western blot analysis of cGAS-STING pathway-related proteins TBK1 and STING in the cerebral cortex (**A**) and hippocampus (**C**) of mice. (**B**,**D**) Quantification of band intensities from the cerebral cortex (**B**) and hippocampus (**D**) samples. Densitometric analysis was performed with ImageJ (https://imagej.org/) and normalized to GAPDH, n = 6 mice per group. All the bar graphs are represented by mean ± SEM; statistical significance was performed with one-way ANOVA followed by Dunnett’s multiple comparisons test. * *p* < 0.05, ** *p* < 0.01, *** *p* < 0.001; ns, not significant. (**E**) Representative confocal immunofluorescence images showed co-staining of the microglial marker P2ry12 (green) and the phosphorylated TBK1 (pTBK1) protein (red) in mouse brain sections, scale bars = 50 μm (left) or 10 μm (right). (**F**) Quantification of pTBK1 immunofluorescence intensity colocalized with P2ry12, n = 6 mice per group. All the bar graphs are represented by mean ± SEM; statistical significance was performed with one-way ANOVA followed by Dunnett’s multiple comparisons test, * *p* < 0.05. Veh, vehicle; D-gal, D-galactose; Res, resveratrol.

To further determine whether resveratrol modulated cGAS-STING signaling in the aging brain, we examined key pathway components in distinct brain regions. Western blot analysis showed that the phosphorylated STING (pSTING) levels were significantly elevated in aging model mice compared with vehicle-treated controls (Figure 5A–D). Resveratrol treatment markedly reduced pSTING expression toward levels observed in control mice. We next evaluated phosphorylated TBK1 (pTBK1), a downstream effector of activated STING. Aging mice exhibited pronounced increases in pTBK1 relative to controls. Resveratrol administration substantially lowered pTBK1 levels in both the cortex and hippocampus (Figure 5A–D). Notably, the cortex showed larger age-related increases in pSTING and pTBK1 than the hippocampus, consistent with regional vulnerability to aging processes. Immunofluorescence staining further supported these findings. Compared with aging controls, resveratrol-treated mice displayed reduced pSTING and pTBK1 immunoreactivity in affected brain regions (Figure 5E,F). Therefore, these findings show that aging-related activation of the cGAS-STING pathway occurs in the mouse brain and can be effectively inhibited by resveratrol. This indicates that inhibition of cGAS-STING signaling may represent a key mechanism by which resveratrol mitigates aging-related neuroinflammatory responses.

### 3.6. Resveratrol Inhibited cGAS-STING Pathway Activation in Microglial Cells

**Figure 6:**
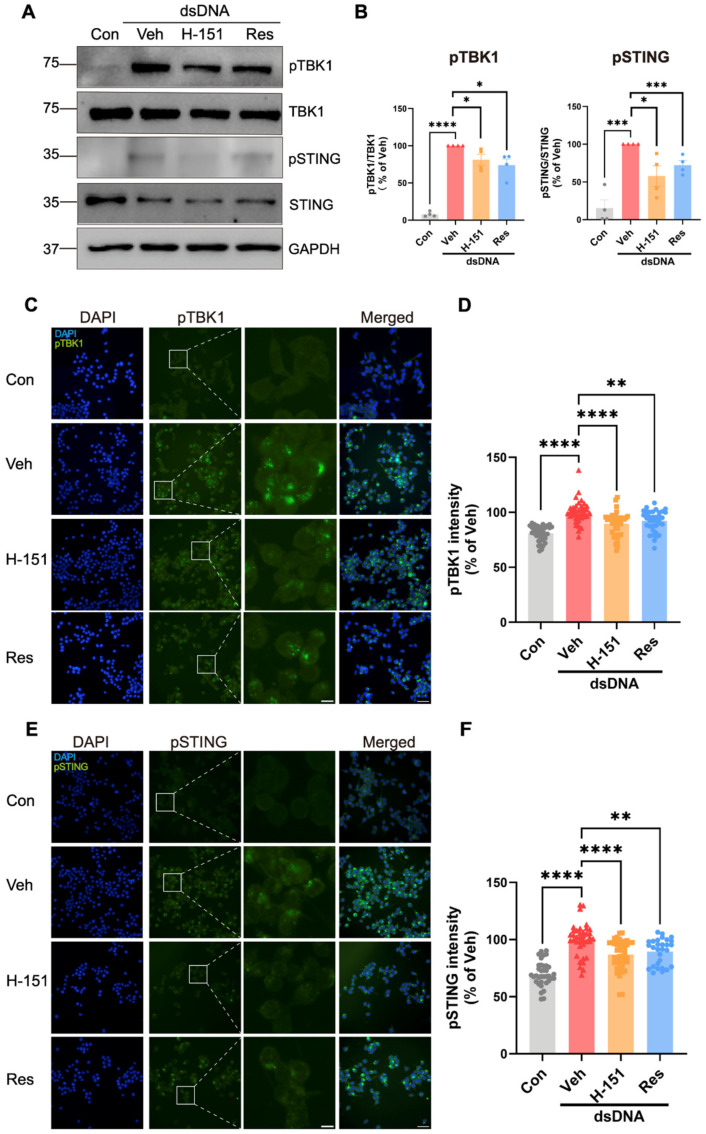
Resveratrol suppressed STING activation in microglial cells. (**A**) Western blot analysis to assess cGAS-STING pathway proteins TBK1 and STING in BV-2 murine microglia. Cells were collected 9 h after dsDNA induction. Prior to induction, cells were pre-treated with resveratrol (Res) or the STING antagonist H-151 for 1 h. (**B**) Quantification of Western blot band intensities. Densitometric analysis was performed using ImageJ and normalized to GAPDH, n = 4 biological replicates. All the bar graphs are represented by mean ± SEM; statistical significance was performed with one-way ANOVA followed by Dunnett’s multiple comparisons test, * *p* < 0.05, *** *p* < 0.001, **** *p* < 0.0001. (**C**,**E**) Representative immunofluorescence images of pTBK1 (**C**) or pSTING (**E**) in BV-2 cells, scale bars = 10 μm (left) or 50 μm (right); (**D**,**F**) Quantification of fluorescence intensity for pTBK1 (**D**) and pSTING (**F**) in BV-2 cells, n = 29–42 images from three independent biological replicates. All the bar graphs are represented by mean ± SEM; statistical significance was performed with one-way ANOVA followed by Dunnett’s multiple comparisons test, * *p* < 0.05, ** *p* < 0.01, *** *p* < 0.001, **** *p* < 0.0001. Con, control; Veh, vehicle; H-151, a selective and covalent STING antagonist; Res, resveratrol; dsDNA, double-stranded DNA.

To extend the in vivo findings of resveratrol-mediated attenuation on cGAS-STING signaling in brain tissue, we next investigated the effects of resveratrol at the cellular level. In these experiments, dsDNA was used to induce STING pathway activation, and we subsequently assessed the effect of resveratrol on the phosphorylation levels of key signaling proteins following activation. Western blot analysis showed that resveratrol treatment effectively inhibited the activation of the STING signaling pathway induced by dsDNA in BV-2 cells (Figure 6A,B). Specifically, the phosphorylation levels of STING and its key downstream protein TBK1 were significantly increased after dsDNA induction, but they were significantly reduced by resveratrol treatment, indicating that resveratrol effectively inhibited STING and its downstream signaling pathway (Figure 6A,B). This inhibition resembled the effect of the STING inhibitor H-151, indicating that resveratrol effectively attenuates the STING-mediated signaling pathway. Upon STING pathway activation, STING oligomerizes and recruits TBK1, leading to TBK1 undergoing autophosphorylation (pTBK1), and in turn phosphorylates STING (pSTING) on key serine residues (e.g., S366), creating a docking site for interferon regulatory factor 3 [13]. These phosphorylated species show pronounced punctate perinuclear staining rather than the diffuse endoplasmic reticulum (ER) and cytosolic pattern seen in resting cells [43]. Thus, we further employed immunofluorescence staining to visualize the spatial distribution and cellular localization of the two key signaling proteins in situ. Interestingly, there was obvious punctate perinuclear staining of pTBK1 and pSTING in the dsDNA-treated cells, instead of diffuse ER localization seen in resting cells (Figure 6C,E). Conversely, both resveratrol and H-151 treatment significantly suppressed pSTING and pTBK1 signal intensity and altered their subcellular distribution induced by dsDNA (Figure 6C–F). Thus, these results corroborate our prior transcriptomic data identifying the cGAS-STING pathway as a major altered signaling cascade in this cell model.

### 3.7. Resveratrol Blocked STING Translocation and Signal Transduction

**Figure 7:**
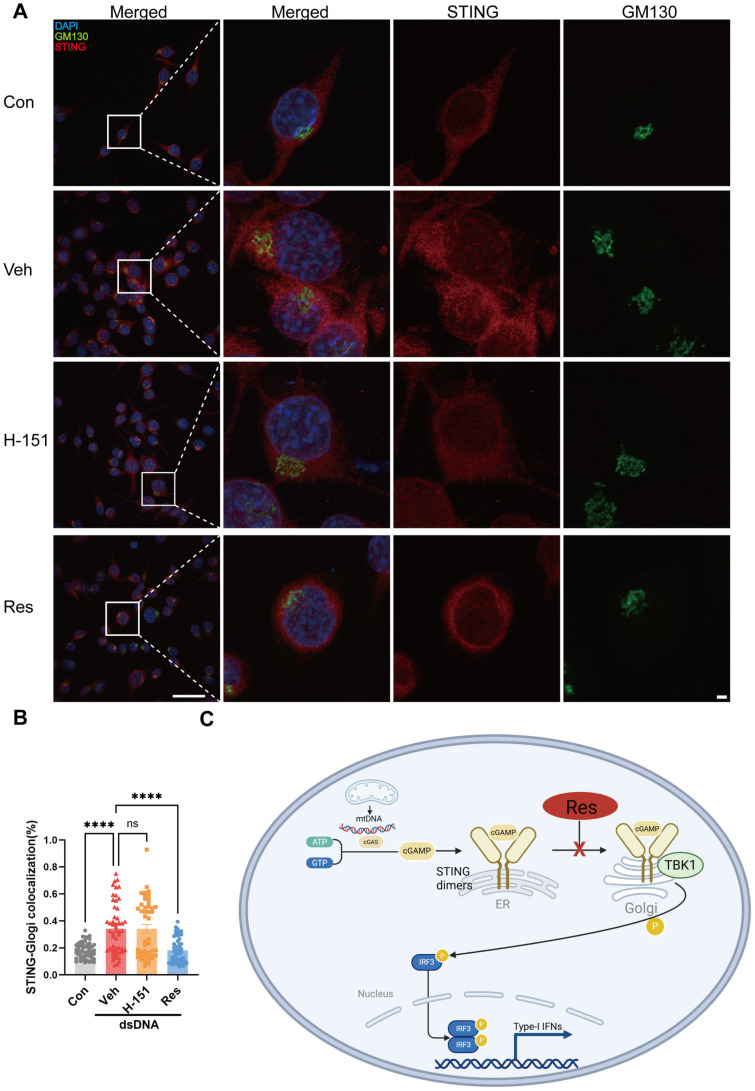
Resveratrol blocked STING translocation to the Golgi apparatus. (**A**) Representative confocal immunofluorescence images showed localization of STING (red) and Golgi marker GM130 (green) in BV-2 cells at 2 h after dsDNA induction, which were pre-treated with resveratrol (Res) or the STING antagonist H-151 for 1 h, scale bars = 50 μm (left) or 10 μm (right). (**B**) Quantification of co-localization between STING and GM130, n = 46–64 cells from three independent biological replicates. All the bar graphs are represented by mean ± SEM; statistical significance was performed with one-way ANOVA followed by Dunnett’s multiple comparisons test, **** *p* < 0.0001; ns, not significant. (**C**) Schematic summary of the proposed mechanism by which resveratrol modulates the cGAS-STING signaling pathway. Con, control; Veh, vehicle; H-151, a selective and covalent STING antagonist; Res, resveratrol; dsDNA, double-stranded DNA.

STING acts as a scaffold protein that recruits downstream signaling components upon binding cyclic dinucleotides. Its translocation from the ER to the Golgi apparatus is a defining step in signal propagation. To investigate how resveratrol modulates STING signaling, we examined the subcellular localization of STING relative to the Golgi. Immunofluorescence assays were performed to assess the co-localization of STING with the Golgi marker GM130. In BV-2 cells, two hours of dsDNA stimulation resulted in a pronounced accumulation of STING around the Golgi, as evidenced by increased overlap with GM130 signals (Figure 7A). This finding is consistent with activation of the pathway and the trafficking of STING toward its signaling platform. In contrast, pre-treatment with resveratrol prior to dsDNA stimulation markedly reduced the amount of STING localized to the Golgi apparatus (Figure 7A). Instead, STING immunosignals were more diffusely distributed throughout the cytoplasm, indicating that resveratrol blocked the translocation of STING to the Golgi (Figure 7A,B). This effect on intracellular trafficking was distinct from that of the STING inhibitor H-151, which did not alter STING localization under the same conditions (Figure 7A,B). To further determine whether the inhibitory effect of resveratrol occurs upstream or downstream of STING activation, cells were directly stimulated with the STING agonist 2′,3′-cGAMP to bypass upstream DNA-sensing events. Even under these conditions, resveratrol treatment significantly suppressed STING translocation from the ER to the Golgi (Supplementary Figure S4A,B). Collectively, these findings suggest that resveratrol blocked a critical step in STING signal transduction by preventing its trafficking from the ER to the Golgi (Figure 7C). This observation reinforced our earlier transcriptomic identification of cGAS-STING signaling as a major altered pathway by resveratrol treatment.

## 4. Discussion

In the present study, we demonstrated that resveratrol, a non-flavonoid polyphenol, effectively ameliorated cognitive decline and behavioral impairments in D-galactose (D-gal)-induced aging mice. Importantly, we provide evidence that resveratrol suppressed microglial senescence and attenuated inflammatory responses both in vivo and in vitro, and we identified a mechanistic link with the cGAS-STING signaling pathway. Our data showed that oral resveratrol markedly attenuated brain aging in mice, as evidenced by reduced expression of senescence-associated markers (p21 and SA-β-gal), decreased DNA damage, suppression of SASP secretion, and improvements in multiple functional outcomes. We further elucidated that resveratrol’s anti-senescence effect is mediated, at least in part, by suppressing STING activation and signaling in microglia. This reveals a previously unrecognized mechanism by which resveratrol modulates innate immune senescence.

Our results add to the growing recognition that the cGAS-STING pathway is a central driver of cellular senescence and age-related neuroinflammation [44]. cGAS, the cytosolic DNA sensor, detects aberrant cytosolic DNA arising from genomic damage, mitochondrial dysfunction, or chromatin fragments, and catalyzes production of cGAMP, which binds STING to initiate downstream signaling via TBK1 and NF-κB, culminating in type I interferon and pro-inflammatory gene expression [13]. This cascade is not only a key innate immune response to pathogens, but also a regulator of the SASP and chronic inflammation during aging [45]. Activation of cGAS-STING has been directly linked to SASP induction and to the inflammatory aspects of senescence in many cell types, including glial cells in the brain. Blockade of STING signaling has been shown to suppress SASP and reduce age-related inflammation and functional decline in peripheral organs and the central nervous system (CNS) of aging mice, and to attenuate reactive microglial states associated with cognitive decline [6,46].

In the aged mouse brain, impaired mitophagy and mitochondrial dysfunction lead to the accumulation of cytosolic mitochondrial DNA in microglia, which aberrantly activates the cGAS-STING pathway, thereby promoting inflammatory gene expression and contributing to neurodegeneration [47,48]. This mechanistic link between cytosolic DNA, cGAS activation, and SASP underscores why the cGAS-STING axis represents a crucial regulator of microglial senescence and age-related neuroinflammatory phenotypes.

Consistent with these reports, our data showed that aging mice exhibited pronounced activation of STING signaling in the cerebral cortex, as indicated by elevated phosphorylation of STING and its downstream kinase TBK1. These molecular changes correlate with enhanced expression of senescence markers and impaired cognitive performance. Notably, oral administration of resveratrol significantly reduced phosphorylation of STING and TBK1 in aged cortex, mitigated microglial senescence, and improved behavioral outcomes. In cultured murine microglial cells, resveratrol suppressed STING activation and downstream inflammatory signaling and reduced SASP factor production. Collectively, these findings position the cGAS-STING pathway as a key mediator of microglial senescence and neuroinflammation and demonstrate the anti-aging effects of resveratrol in the brain at least partly via inhibition of STING activation.

A particularly interesting finding from our study is that resveratrol inhibited STING translocation and signaling, suggesting a specific regulatory mechanism beyond general anti-oxidation or anti-inflammation. Notably, this effect on trafficking differed mechanistically from that of the canonical STING inhibitor H-151, which left STING localization unaltered. STING resides on the ER in resting cells, and upon activation it translocates to the ER-Golgi intermediate compartment and Golgi, where it recruits TBK1 and triggers downstream phosphorylation events. Limiting STING translocation is an effective means to block downstream signaling and SASP induction. Although this is the first report to our knowledge of resveratrol acting directly on STING activation or translocation in microglia, similar mechanisms have been described for other modulators of STING that prevent its ER-to-Golgi trafficking [49,50]. Several mechanistic hypotheses may explain the effects of resveratrol on STING signaling. First, resveratrol has been reported to influence cellular membrane dynamics and lipid composition [51,52], which could alter ER-Golgi trafficking and impede STING translocation. Second, by reducing oxidative stress and mitochondrial dysfunction [53,54], resveratrol may limit the accumulation of cytosolic DNA that serves as the ligand for cGAS activation, thereby reducing the initiator signal for this pathway. Third, resveratrol might induce or enhance post-translational modifications of STING or its regulators that disrupt effective complex formation with TBK1, although direct biochemical evidence for such modifications remains to be established. Finally, resveratrol could modulate upstream regulators of ER-Golgi transport or chaperone proteins that facilitate the trafficking of STING, thereby exerting an indirect but functionally significant inhibitory effect on signal propagation. To fully elucidate these mechanisms, future studies should assess whether resveratrol directly interacts with STING or associated proteins, how it affects the post-translational modifications of STING, and whether mitochondrial quality control pathways are involved.

Beyond mechanistic insights, our findings highlight the cGAS-STING pathway as a potential therapeutic target for brain aging. Aberrant activation of this innate immune pathway has been closely linked to age-associated neuroinflammation and microglial dysfunction, processes that contribute to the development and progression of neurodegenerative diseases such as Alzheimer’s and Parkinson’s disease. By showing that resveratrol suppresses cGAS-STING signaling and ameliorates microglial senescence, our study provides evidence that modulation of innate immune pathways may represent a promising strategy for mitigating brain aging and related cognitive decline. Nevertheless, several limitations should be acknowledged. First, the current study relied on a D-galactose-induced aging model, which, although widely used, does not fully replicate the complexity of natural aging. Validation in naturally aged mice will therefore be essential to confirm the physiological relevance of our findings. In addition, future studies should evaluate the effects of resveratrol in neurodegenerative disease models and explore potential combinational strategies with other anti-aging interventions, including senolytics or NAD^+^-boosting therapies.

## 5. Conclusions

In conclusion, our study revealed that resveratrol mitigated brain aging and microglial senescence by suppressing cGAS-STING pathway activation and STING signal transduction. These results not only support the central role of the cGAS-STING axis in microglial senescence and age-related brain dysfunction but also highlight a novel anti-aging mechanism of resveratrol that may be exploited for future therapeutic development. Therefore, targeting this pathway may represent a promising therapeutic strategy for age-related neuroinflammatory and cognitive disorders.

## Data Availability

The original contributions presented in this study are included in the article/Supplementary Materials. Further inquiries can be directed to the corresponding author.

## Supplementary Materials

The following supporting information can be downloaded at: https://www.mdpi.com/article/10.3390/cells15060523/s1, Supplementary Figure S1. Body weight trajectories of aging mice during resveratrol treatment. Changes in body weight of mice from 8 to 16 weeks of age, n = 12 mice per group, data are represented as mean ± SEM; statistical significance was performed with two-way ANOVA followed by Sidak’s multiple comparisons test, ns, not significant. Veh, vehicle; D-gal, D-galactose; Res, resveratrol. Supplementary Figure S2. Effect of resveratrol on markers of DNA damage and cellular senescence in aging mice. (**A**) Representative immunofluorescence images showed co-staining of the microglial marker IBA1 (red) and senescence marker p21 (green) in brain sections of Res-treated aging mice, scale bars = 50 μm (left) or 10 μm (right). (**B**) Quantification of p21 fluorescence intensity colocalized with IBA1. (**C**) Representative confocal immunofluorescence images of mouse brain sections co-stained for 8-OHdG (green) and DAPI (blue); scale bar = 50 μm. (**D**) Quantification of fluorescence intensity for 8-OHdG. (**E**) Representative immunofluorescence images showed co-staining of the neuronal marker TUBB3 (green) and senescence marker β-gal (red) in brain sections of Res-treated aging mice, scale bars = 50 μm (left) or 10 μm (right). (**F**) Quantification of β-gal fluorescence intensity colocalized with TUBB3. N = 6 mice per group, data are represented as mean ± SEM; statistical significance was performed with one-way ANOVA followed by Dunnett’s multiple comparisons test. ** *p* < 0.01, *** *p* < 0.001; ns, not significant. Veh, vehicle; D-gal, D-galactose; Res, resveratrol; β-gal, beta-galactosidase. Supplementary Figure S3. RNA-seq analysis of genes differentially regulated by resveratrol and dsDNA. (**A**) GSEA showed correlation of dsDNA treatment with gene sets associated with the Cytosolic DNA-Sensing Pathway. (**B**) GSEA of gene expression showed upregulation of the Cytosolic DNA-Sensing Pathway in dsDNA-treated BV-2 cells versus vehicle controls. (**C**) GSEA showed association of resveratrol treatment with gene sets involved in the Cytosolic DNA-Sensing Pathway. (**D**,**E**) GO analysis showed alterations in biological process (**D**) and cellular component (**E**) in Res-treated BV-2 cells. (**F**) Venn diagram of overlapping differentially expressed genes (DEGs) that are upregulated by resveratrol or downregulated by dsDNA. (**G**) Heatmap of RNA sequencing data depicting genes that are downregulated following dsDNA induction but upregulated upon resveratrol treatment. Veh, vehicle; D-gal, D-galactose; Res, resveratrol; β-gal, beta-galactosidase. Supplementary Figure S4. Resveratrol blocked STING translocation to the Golgi apparatus. (**A**) Representative confocal immunofluorescence images show localization of STING (red) and Golgi marker GM130 (green) in BV-2 cells at 1 h after cGAMP induction, which were pre-treated with resveratrol (Res) for 1 h, scale bars = 50 μm (left) or 10 μm (right). (**B**) Quantification of co-localization between STING and GM130, n = 41–77 cells from three independent biological replicates. All the bar graphs are represented by mean ± SEM; statistical significance was performed with one-way ANOVA followed by Dunnett’s multiple comparisons test, **** *p* < 0.0001. Veh, vehicle; cGAMP, 2’, 3’-cyclic GMP-AMP; Res, resveratrol. Table S1. Reagents and primers used in this study.

## Abbreviations

The following abbreviations are used in this manuscript:

cGAS	cyclic GMP-AMP synthase
STING	stimulator of interferon genes
SASP	senescence-associated secretory phenotype
D-gal	D-galactose
Cxcl-10	C-X-C motif chemokine ligand 10
Il-1β	interleukin 1 beta
TBK1	TANK binding kinase 1
AD	Alzheimer’s disease
PD	Parkinson’s disease
P16	cyclin-dependent kinase inhibitor 2A
P21	cyclin-dependent kinase inhibitor 1A
2′, 3′-cGAMP	2′, 3′-cyclic GMP-AMP
CMC-Na	sodium carboxymethylcellulose
PBS	phosphate-buffered saline
pTBK1	phosphorylated TBK1
IBA1	induction of brown adipocytes 1
P2ry12	purinergic receptor P2Y, G-protein coupled 12
TUBB3	Tubulin beta 3
CD68	cluster of differentiation 68
DAPI	4′,6-diamidino-2-phenylindole
GM130	golgi autoantigen
SA-β-gal	senescence-associated β-galactosidase
SDS-PAGE	sodium dodecyl sulfate polyacrylamide gel electrophoresis
pSTING	phosphorylated STING
GAPDH	glyceraldehyde-3-phosphate dehydrogenase
FBS	fetal bovine serum
dsDNA	double-stranded DNA
DMSO	dimethyl sulfoxide
ELISA	enzyme-linked immunosorbent assay
TNF-α	tumor necrosis factor alpha
cDNA	complementary DNA
RT-qPCR	real-time quantitative PCR
DEGs	differentially expressed genes
GO	gene ontology
KEGG	Kyoto Encyclopedia of Genes and Genomes
SEM	standard error of the mean
CA1	cornu ammonis 1
DG	dentate gyrus
8-OHdG	8-hydroxy-2′-deoxyguanosine
CA3	cornu ammonis 3
EC	entorhinal cortex
Ccl-2	C-C motif chemokine ligand 2
Ifi-44	interferon-induced protein 44
Il-18	interleukin 18
GSEA	gene set enrichment analysis
ER	endoplasmic reticulum
CNS	central nervous system
NAD	nicotinamide adenine dinucleotide
