# Supplementary material for: Resveratrol Mitigates Age-Associated Cognitive Decline via Inhibition of cGAS-STING-Mediated Microglial Senescence
Source: Cells. 2026 Mar 16;15(6):523. doi: 10.3390/cells15060523 (PMC13025151; doi:10.3390/cells15060523)
Supplement: Supplementary file 1 [file cells-15-00523-s001.zip › supplementary materials.pdf]

Supplementary Materials

Title: Resveratrol mitigates age-associated cognitive decline via inhibition of cGAS-STING mediated microglial senescence

Materials and Methods.

2′,3′-cGAMP treatment. 2′,3′-cGAMP (MeilunBio, #MC13179-1) stock solutions were prepared in PCR-grade water at 10mg/ml, respectively, and stored at -20 °C. Working solutions of 2′,3′-cGAMP (2 μg/ml) was freshly prepared in DMEM prior to use. Cells were pretreated with resveratrol for 1 h. Following pretreatment, 2′,3′-cGAMP was added and cells were co-treated for 1 h with the original compounds present.

Supplementary Figure

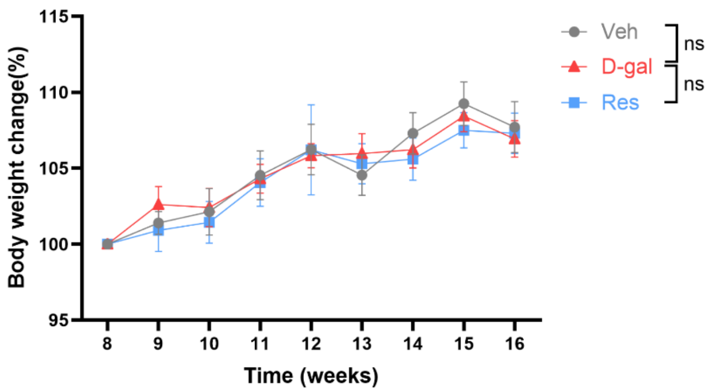

Supplementary Figure S1. Body weight trajectories of aging mice during resveratrol treatment. Changes in body weight of mice from 8 to 16 weeks of age, n=12 mice per group, data are represented as mean ± SEM; statistical significance was performed with two-way ANOVA followed by Sidak's multiple comparisons test, ns, not significant. Veh, vehicle; D-gal, D-galactose; Res, resveratrol.

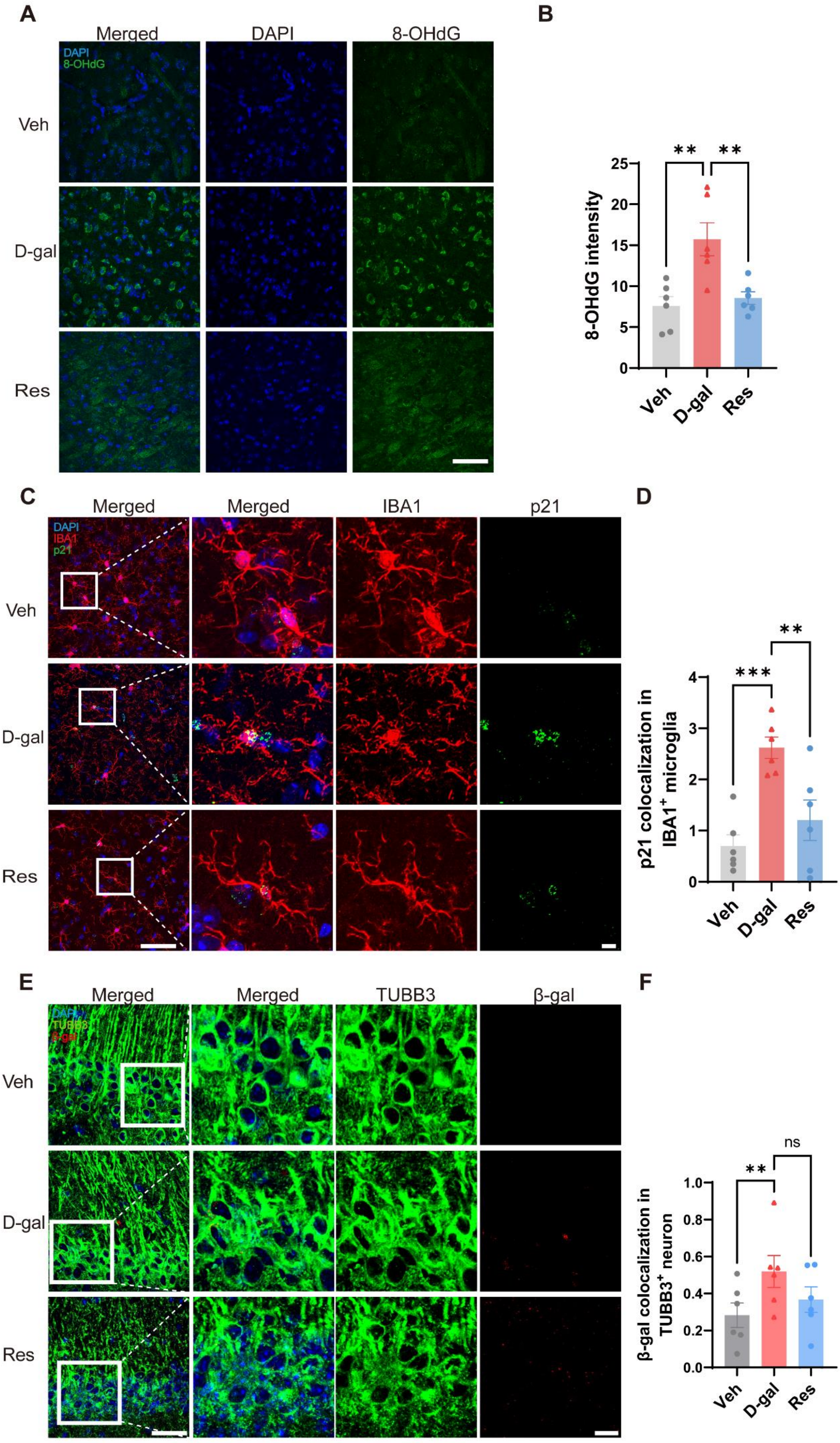
A
Merged
DAPI
8-OHdG
DAPI
8-OHdG
Veh
D-gal
Res
B
8-OHdG intensity
**
**
25
20
15
10
5
0
Veh
D-gal
Res
C
Merged
Merged
IBA1
p21
DAPI
IBA1
p21
Veh
D-gal
Res
D
p21 colocalization in IBA1⁺ microglia
***
**
4
3
2
1
0
Veh
D-gal
Res
E
Merged
Merged
TUBB3
β-gal
DAPI
TUBB3
β-gal
Veh
D-gal
Res
F
β-gal colocalization in TUBB3⁺ neuron
**
ns
1.0
0.8
0.6
0.4
0.2
0.0
Veh
D-gal
Res

Supplementary Figure S2. Effect of resveratrol on markers of DNA damage and cellular senescence in aging mice. A, Representative immunofluorescence images showed co-staining of the microglial marker IBA1 (red) and senescence marker p21 (green) in brain sections of Res-treated aging mice, scale bars=50μm (left) or 10μm (right). B, Quantification of p21 fluorescence intensity colocalized with IBA1. C, Representative confocal immunofluorescence images of mouse brain sections co-stained for 8-OHdG (green) and DAPI (blue); scale bar = 50μm. D, Quantification of fluorescence intensity for 8-OHdG.E, Representative immunofluorescence images showed co-staining of the neuronal marker TUBB3 (green) and senescence marker β-gal (red) in brain sections of Res-treated aging mice, scale bars=50μm (left) or 10μm (right). F, Quantification of β-gal fluorescence intensity colocalized with TUBB3. N=6 mice per group, data are represented as mean ± SEM; statistical significance was performed with one-way ANOVA followed by Dunnett's multiple comparisons test. **$p<0.01$; ns, not significant. Veh, vehicle; D-gal, D-galactose; Res, resveratrol; β-gal, beta-galactosidase.

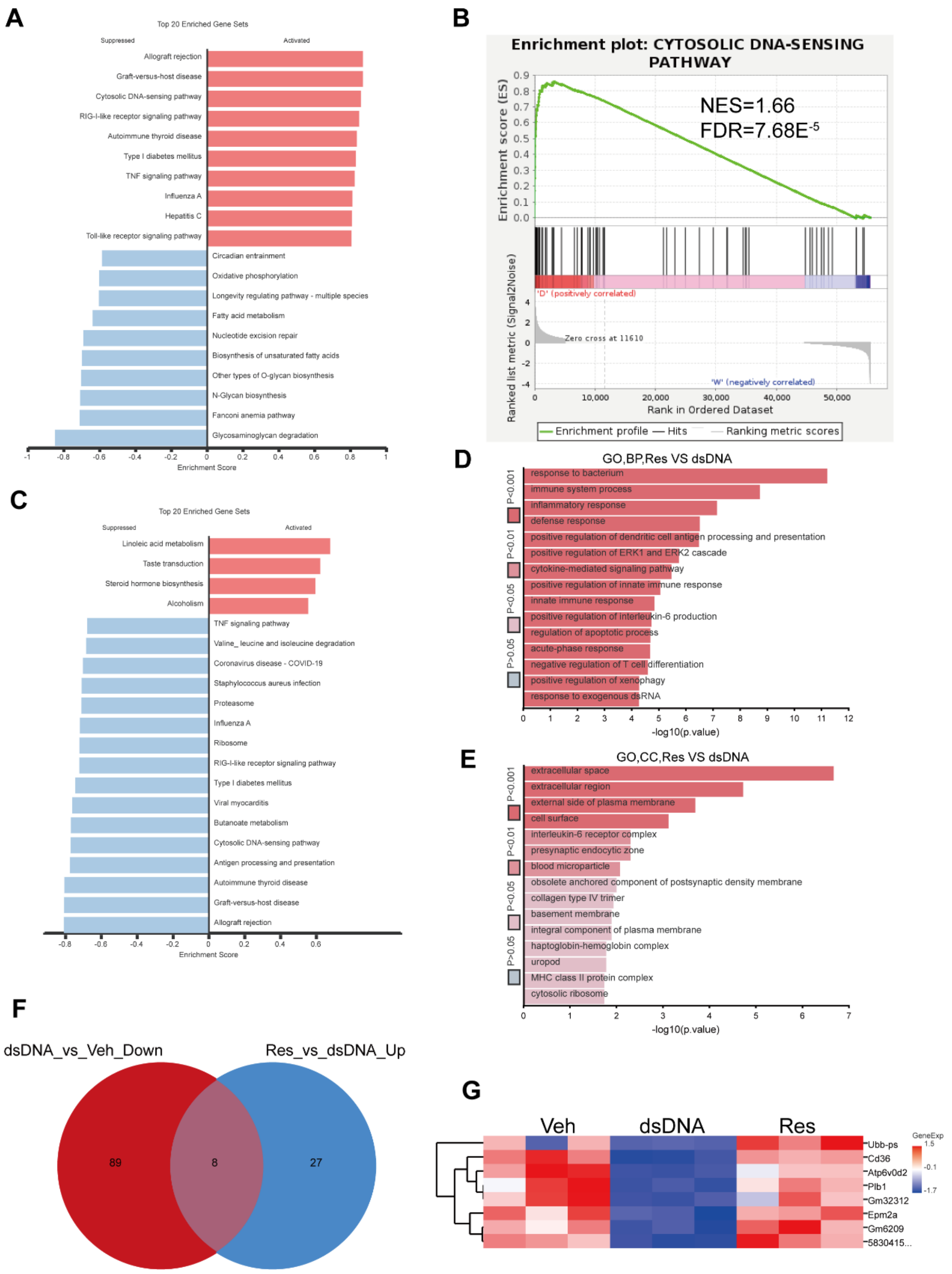

Supplementary Figure S3. RNA-seq analysis of genes differentially regulated by resveratrol and dsDNA. A, GSEA analysis showed correlation of dsDNA treatment with gene sets associated with the cytosolic DNA-sensing pathway. **B,** GSEA analysis of gene expression

showed upregulation of the cytosolic DNA-sensing pathway in dsDNA-treated BV-2 cells versus vehicle controls. **C**, GSEA analysis showed association of resveratrol treatment with gene sets involved in the cytosolic DNA-sensing pathway. **D** and **E**, GO analysis showed alterations in biological process (**D**) and cellular component (**E**) in Res-treated BV-2 cells. **F**, Venn diagram of overlapping differentially expressed genes (DEGs) that are upregulated by resveratrol or downregulated by dsDNA. **G**, Heatmap of RNA sequencing data depicting genes that are downregulated following dsDNA induction but upregulated upon resveratrol treatment. Veh, vehicle; D-gal, D-galactose; Res, resveratrol; β-gal, beta-galactosidase.

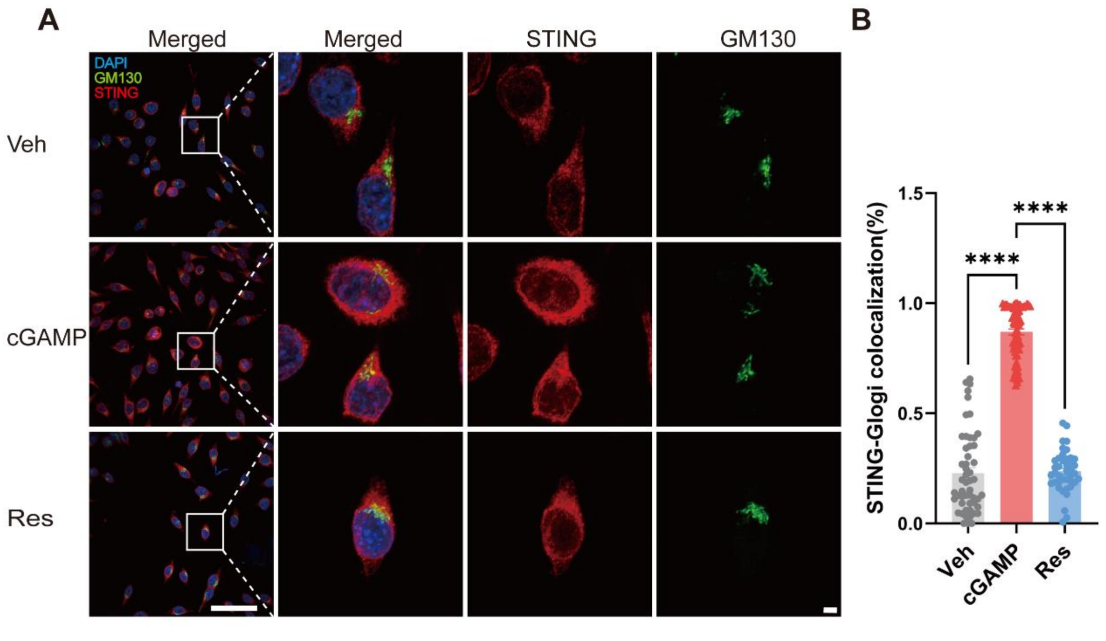

Supplementary Figure S4. Resveratrol blocked STING translocation to the Golgi apparatus. A, Representative confocal immunofluorescence images showed localization of STING (red) and Golgi marker GM130 (green) in BV-2 cells at 1h after cGAMP induction, which were pre-treated with resveratrol (Res) for 1 h, scale bars=50μm (left) or 10μm (right). B, Quantification

of colocalization between STING and GM130, n=41-77 cells from three independent biological replicates. All the bar graphs are represented by mean ± SEM; statistical significance was performed with one-way ANOVA followed by Dunnett's multiple comparisons test, ****$p < 0.0001$. Veh, vehicle; cGAMP, 2′, 3′-cyclic GMP-AMP; Res, resveratrol.
